# Gross motor skills trajectory variation between WEIRD and LMIC countries: A cross-cultural study

**DOI:** 10.1371/journal.pone.0267665

**Published:** 2022-05-05

**Authors:** Nadia Cristina Valentini, Glauber Carvalho Nobre, Marcelo Gonçalves Duarte

**Affiliations:** 1 Department of Physical Education, Physiotherapy, and Dance, Universidade Federal do Rio Grande do Sul, Porto Alegre, Brazil; 2 Department of Physical Education and Sports, Instituto Federal do Ceará, Fortaleza, Ceará, Brazil; 3 Department of Physical Education, Universidade Federal do Mato Grosso do Sul, Corumbá, Brazil; The Education University of Hong Kong, HONG KONG

## Abstract

**Aim:**

This study aimed to examine the prevalence of delays and borderline impaired performance for Brazilian girls and boys and the differences in the motor trajectories (locomotor and ball skills) of girls and boys (3- to 10-years-old) across WEIRD (Western, Educated, Industrial, Rich, and Democratic) countries and Brazil–a low- and middle-income country (LMIC).

**Methods:**

We assessed 1000 children (524 girls; 476 boys), 3- to 10.9-year-old (M = 6.9, SD = 2.1; Girls M = 6.9, SD = 2.0; Boys M = 6.9, SD = 2.1), using the Test of Gross Motor Development-3. Using systematic search, original studies investigating FMS in children using the TGMD-3 were eligible; 5 studies were eligible to have the results compared to the Brazilian sample. One sample t-test to run the secondary data from Irish, American, Finnish, and German children (i.e., mean, standard deviation).

**Results:**

The prevalence of delays and borderline impaired performance was high among Brazilian girls (28.3% and 27.5%) and boys (10.6% and 22.7%). The cross-countries comparisons showed significant (p values from .048 and < .001) overall lower locomotor and ball skills scores for Brazilian children; the only exceptions were skipping, catching, and kicking. We observed stability in performance, across countries, after 8-years-old, and no ceiling effects were found in the samples.

**Conclusions:**

The Brazilian sample emphasized the need for national strategies to foster children’s motor proficiency. Differences in motor opportunities may explain the differences in motor trajectories between children in WEIRD and LMIC countries.

## Introduction

During childhood, motor proficiency is hugely variable from child to child. Differences in individual characteristics (e.g., body mass index, age, sex [1–5]; and opportunities to practice skills [6–10] are often reported as relevant factors in those disparities. In addition, caregivers support [11, 12], population density [4], cultural environment [13–15], geographic regions [14, 16, 17], and socioeconomic status [16, 18, 19] also affects children’s motor proficiency.

Motor proficiency is also variable across countries due to cultural experiences; the evidence was reported for Belgium and Australia [20]; the United States [18, 21] and Greece [8]; Portugal and the United States [22]; and Greece, Italy, and Norway [23]. Of these studies, D’ Hondt et al. [8] provided evidence that Belgium and Greek preschool children had similar gross motor proficiency. Bardid and colleagues [21] reported that Belgian children scored higher in three motor tasks (i.e., jumping, moving sideways, hopping) than 6- to 8-years-old Australian children and lower in object control skills than the 3- to-7-years-old American children. The authors suggested that differences in physical education programs in European countries and across continents related to sports practice—specifically in striking with a bat and overarm throwing—play an essential role in the results.

Regarding cross-cultural comparisons among WEIRD (Western, Educated, Industrial, Rich, and Democratic) and LMIC (Low- and Middle-Income Countries) countries, the literature is more robust regarding young ages (i.e., infants and toddlers). Overall higher gross motor scores are reported for Canadians [15, 24–26] and Greek [15] infants compared to Brazilians; and American and German compared, respectively, to Malawian [27] and Cameroonian Nso [28] infants. These results indicate culturally specific differences concerning tests tasks—infants perform higher in the tasks closer to their familiar experiences, parental beliefs regarding childrearing, and opportunities for movement explorations [15, 24, 25, 27–29].

For preschoolers and school-age children, one recent study compares the motor proficiency of children (4 to 11 years old) from the south of Brazil and Portugal. Brazilian boys’ scores were above Portuguese normative values in four out of six tasks (i.e., long jump, kick and throw velocity, shuttle run), and Brazilian girls performed below those values in all but one task (i.e., long jump). The authors suggested that differences in the physical education curriculum, children’s free time, and opportunities may explain these results [30]. These results are, to some extent, not expected since previous research reported a high prevalence of delays among Brazilian children [19, 31–36], in studies with included larger samples and several states. Overall, the delays have been related to socioeconomic restrictions [31, 32, 36, 37] and the consequently limited resources at home [38] and schools [14] to meet the children’s motor developmental needs.

These previous differences across countries were established using mostly the TGMD-2. To the authors’ knowledge, no studies also compared the children’s motor proficiency across countries using the TGMD-3. The TGMD-3 has included two new skills—skip and one-hand strike, besides two skills (two-hands strike and kick) that have been part of the assessment since its early version. These four skills could present a more robust cultural component interfering with the childhood motor trajectories–the practice of specific sports. The one-hand strike is practiced in tennis, although an increasing number of younger children has become involved with this sport, it mainly occurs in the United States and European countries [39]; in Brazil, the sport is still elitist with a high cost to participate [40]. Consequently, Brazilian children are less exposed to the practice of this skill. In addition, the two-hands strike, and the kick are highly practiced in the United States and Latin American countries, respectively, due to the baseball/softball and soccer culture; if it transfers to a different motor trajectory it is not known.

The opportunities for children to become motor proficiency (e.g., physical education curriculum, engagement in physical education lessons, and enrollment in after-school sports programs) in WEIRD countries are not like the one found for children in the LMIC(s); nor are the results of research in WEIRD countries directly translate to children living in other contexts. Cross-cultural information is relevant to promoting motor proficiency, physical activity, and health outcomes among children [8]. Furthermore, comparing the gross motor skills trajectories across a wide age range for boys and girls from WEIRD and LMIC countries may provide new insights to understand the periods of maximum acquisition and stability in children’s performance in different cultures. This information may be essential to promote motor activity engagement across childhood and support the development of policies for monitoring motor proficiency and delays among children—common goals for researchers in different countries but highly relevant for low-income populations.

Moreover, a large population-based study that enrolled preschool and school-age children of a wide range of ages and from all regions of Brazil can lead to a deep comprehension of motor proficiency and the prevalence of motor delays in children. Specifically, the large age band could better represent the periods that occur more discontinuity in motor development, allowing for specific strategies to prevent the high rates of delay reported previously; and the enrollment of all regions could provide representatives regarding cultural differences in a continental country. Therefore, this study aimed to examine the prevalence of delays and borderline performance for Brazilian girls and boys and the differences in the motor trajectories (locomotor and ball skills) of girls and boys (3- to 10-years-old) across WEIRD countries and an LMIC country–Brazil. These objectives were grounded on the understanding that the identification of children with delays and borderline performance in LMIC(s) is crucial to providing adequate care and that cross-cultural research across European, North American, and Latin American countries would illustrate the range of cultural variation of motor practices that could affect motor trajectories. Besides, grounded on the knowledge that differences in culture influence how children acquire and practice motor skills, the age and sequence in which children acquire them, and the subsequent developmental trajectory [41], this study examined the variation in the trajectories at a particular skill for girls and boys across the countries.

## Methods

This study was a part of a national study on children’s gross motor skills performance, and it is organized into two sections: a systematic review and a field study with Brazilian children.

### Systematic review: Procedures and data extraction

**Fig 1:**
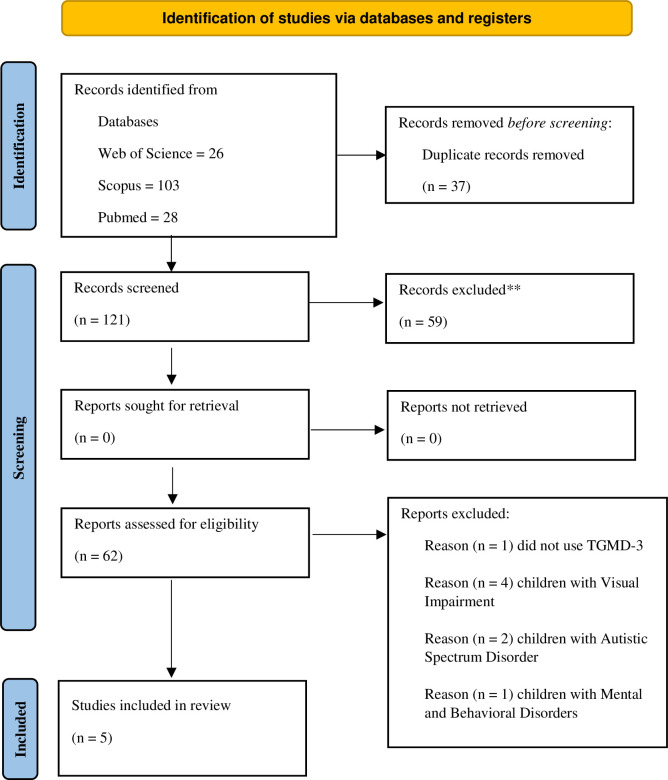
PRISMA flow diagram for the systematic review.

We included studies using a systematic search procedure to select the studies for the countries’ comparisons. All the original studies investigating fundamental motor skills in children using the TGMD-3 were eligible. We used the PECOT (Population, Exposure, Comparing, Outcome, and Type of study) following the PRISMA protocol as an auxiliary method to include studies [42]. Therefore, we included studies conducted with children from 3- to 10-years-old (Population) assessed with the TGMD-3 (Exposure) and that present results for locomotor and ball skills (Outcome) reported in observational peer-reviewed journals (Type of study). Besides, we included the TGMD-3 manual (US sample). We excluded studies that did not report locomotor or ball skills scores according to age and sexes, at least in three age groups. Fig 1 presents the PRISMA flow diagram for the systematic review.

Following the PRISMA protocol, we conducted a computerized search using Web of Science, MEDLINE (accessed by PubMed), and Scopus databases. We utilized the logic-based specific descriptors, Boolean operators (AND & OR), and parentheses. We used the following combination of keywords to locate studies from database inception to September 2020: "children" AND "child" or "childhood," AND "fundamental motor skill" OR "fundamental motor skills" OR "fundamental movement skills" OR "fundamental movement skill" OR "motor ability" OR "motor competence" OR "motor proficiency" OR "movement skills" OR "movement skill" AND "test of gross motor development third edition" OR "TGMD-3". We did not add filters (e.g., language, publication date, target audience).

We conducted the exportation of the articles with the Medline, Ris, and Bibtex extensions. We imported the data using the software for systematic review StArt (State of the Art through Systematic Review) to identify duplicated, exclusion, and inclusion articles. Two co-authors (MGD and GCN) conducted this procedure. Studies’ titles and or abstracts retrieved using the search strategy and those from additional sources were screened independently by two authors (MGD and GCN) to identify studies that potentially meet the inclusion criteria; the same co-authors retrieved and independently assessed the studies for full eligibility. We solved the disagreements over the eligibility of studies by a third reviewer (MSS). Finally, we removed the duplicated studies and those that failed to meet inclusion criteria.

Through database researching we identified 157 papers (Web of Science = 26; Scopus = 103; Pubmed = 28). After removing 37 duplicates, we screened 120 studies by title and abstract, 61 studies were eligible for full-text screening. After the full-text screening, four articles matched the review criteria. Besides, we included the TGMD-3 manual manually. Thus, we included five studies conducted within the last five years—all developed economies (Germany, Finland, Ireland, and the United States), besides Brazil’s present study.

The samples included children from Germany [43], Finland [4, 44], Ireland–study 1 [3] and study 2 [2], and the United States—US [45]. The German study did not include the stationary two-hands strike in ball skills. The study 2 on Irish children did include a new locomotor skill, the vertical jump [2]. Therefore, we did not include the German study’s ball skills scores [43] and the locomotor scores from the Irish study 2 [2]. We extracted the selected studies’ locomotor skills and ball skills means and standard deviations. Therefore, we conducted the study across two mainly diverse contexts, Brazil—an LMIC country, and compared the Brazilian data with five WEIRD countries.

### Field study: Participants, instruments, procedures, and data analysis

#### Participants

Participants were 1000 children (girls n = 524; boys n = 476), 3 to 10.9 years old (M = 6.9, SD = 2.1; Girls M = 6.9, SD = 2.0; Boys M = 6.9, SD = 2.1), residing in five main geographic regions of Brazil (North, Northeast, Central-West, Southeast, and South) including eight states (Amazonas, Pará, Ceará, Goiás, Mato Grosso do Sul, Minas Gerais, Santa Catarina, Rio Grande do Sul), ten cities, and 22 schools (public and private). The sample size was calculated in EpiInfo statistical software (version 7.0), considering an approximate population of 800,000 children, an expected 50% frequency, a design effect of 1.5, a 95% confidence level, an 4% acceptable margin of error, and 10 to 15% of possible losses. A final estimated sample necessary was between 990 and 1035 children. The sample was representative of the Brazilian population regarding the children attending public (92%) and private (8%) schools, (b) children attending pre-school (3 to 5 years old: 41%), and fundamental-school (6 to 10 years old: 59%), (c) family’s socioeconomic status (SES) (approximately 10% middle-high and high, 30% middle, and 60% middle-low and poor), (d) children’s biological sex (girls 52.4%; boys 47.6%), and country regions [46]. The exclusion criterium was children with disabilities (i.e., cerebral palsy, motor disorders, intellectual and psychiatric disabilities) reported by parents, teachers, or caregivers.

#### Instrument

We used the Test of Gross Motor Development - 3rd Edition (TGMD-3) [45] validated for Brazilian children [47] to assess children’s locomotor skills (LOCS: run, gallop, hop, skip, horizontal jump, slide) and ball skills (BS: two-hands strike, one-hand strike, stationary dribble, catch, kick, overhand throw, underhand throw) performance. Each skill has 3 to 5 performance criteria describing the efficient movement pattern. We assess the children using the TGMD-3 protocol recommendation. Following the TGMD-3 manual, we used the raw score for each subtest (LOC: 0 to 46; BS: 0 to 54) and the sum of the subtest to convert the scores in the Gross Motor Index (GMI) to obtain the categorization of motor delays (GMI scores < 70) and borderline impaired performance (GMI scores < 70 and 79) in the present study [45].

#### Procedures

The university ethics committee approved the study (CA 2008018). We contacted the board of education and school administrators from several cities in different states of Brazil; 22 schools in eight states responded positively to participation and agreed that we conducted the assessment in the school facilities. Researchers explained the purposes and procedures of the study in a meeting with the school administrator, staff, and teachers. We randomly selected children and parents within the school, or legal guardians were contacted and received all information about the research procedures. Writing consent was obtained from each child’s parents or custodial caregiver(s); each child verbally agreed to participate in the study. Trained professionals assessed the children consecutively, in pairs at schools. The assessments occur during one session of approximately 30 minutes. All tests were video recorded for later motor coding.

Data quality control followed a two-step process. First, two trained raters with extensive coding experience with the TGMD-3 test coded all video records (N = 1000). Then, 100 children were randomly selected for intra-rater and interrater reliability analysis. ANOVA-based intraclass correlations were conducted. Intra-rater (with two-month interval; ICC = from .70 to .90) and interrater (ICC = .85 to .99) reliability were high. Second, data entry errors and inconsistent records were identified and corrected. Data cleaning was performed in Microsoft Excel—360 and IBM SPSS, version 22.0 (SPSS Inc., Chicago, IL, US).

#### Data analysis

Shapiro Wilk test was used to verify the normality of data distribution according to each tested condition (e.g., boys and girls by age); the results showed normality of data distribution in all conditions (values between .963 and .071). Descriptive analyses (i.e., mean, standard deviation, absolute frequency, and percentage) were conducted. We used the One-Sample t-tests to compare the motor performance (LOCS and BS skills) of Brazilian children with the performance of the children from other countries’ samples, Ireland [2, 3]; Germany [43]; Finland [4, 44], and the US [45]. Cohen’s d analyses were provided as a measure of effect size. We conducted all analyses using Statistical Package for Social Science–SPSS, version 22 for Windows (SPSS Inc., Chicago, IL, US); we adopted the significance level at α < 0.05.

## Results

### Motor delays and borderline impaired performance for Brazilian girls and boys

Table 1 provides the means (M) and standard deviations (SD) for the LOCS and BS and the prevalence of delays and borderline performance for Brazilian boys and girls by age. The prevalence of delays (girls: 28.3%; boys: 10.6%) and borderline impaired (girls: 27.5%; boys 22.7%) performances were high for girls and boys. Delays increased with age for boys and girls, and borderline performance increased for girls from 3 to 6 years old and remained around 20 percent until 10-years-old; whereas for the boys, it increased until the age of 7-years-old and then remained stable at around 30 percent.

**Table 1:** Brazilian sample: Mean (M) and standard deviation (SD) of the motor skills performance of children by age (N = 1000).

	GIRLS					BOYS				
Age (years)	Raw Scores and Prevalence of Delays & Borderline					Raw Scores & Prevalence of Delays & Borderline				
	N	LOCS M (SD)	BS M (SD)	Delays N (%)	Borderline N (%)	N	LOCS M(SD)	BS M(SD)	Delays N(%)	Borderline N (%)
**3**	28	13.7 (7.6)	13.1 (4.3)	1 (2.7)	9 (24.3)	20	13.5 (6.1)	16.4 (4.6)	-	3 (7.1)
**4**	61	19.9 (7.3)	18.3 (6.3)	-	9 (18.8)	54	19.2 (6.8)	21.7 (7.0)	-	2 (4)
**5**	81	22.7 (5.9)	19.2 (5.6)	2 (2.9)	23 (33.8)	86	23.4 (6.6)	24.6 (7.1)	1 (1.4)	6 (8.7)
**6**	70	25.4 (7.2)	22.0 (6.6)	15 (22.4)	29 (43.3)	61	27.2 (5.7)	30.2 (6.2)	2 (2.5)	11 (13.8)
**7**	77	28.8 (6.6)	27.2 (7.1)	20 (27.0)	21 (28.4)	51	30.0 (7.2)	33.5 (7.7)	2 (3.4)	22 (37.9)
**8**	73	29.4 (6.7)	30.0 (9.2)	30 (42.9)	15 (21.4)	55	29.6 (6.7)	36.2 (8.1)	14 (19.4)	22 (30.6)
**9**	69	30.6 (6.2)	31.3 (8.6)	38 (51.4)	17 (23.0)	89	30.7 (6.4)	36.8 (7.6)	14 (17.9)	31 (39.7)
**10**	65	30.1(5.6)	29.9 (7.0)	35 (58.3)	14 (23.3)	60	31.4 (5.3)	36.1 (7.8)	20 (37.7)	17 (32.1)
**Total**	524	24.7 (8.6)	24.5 (9.8)	141 (28.3)	137 (27.5)	476	25.0 (8.6)	28.2 (9.0)	53 (10.6)	114 (22.7)

Note: M: mean; SD: standard deviation; LOCS: Locomotor skills; BS: Ball skills; GMQ: Gross motor quotient; Raw score range: LOCS = 0 to 46, BS = 0 to 54; Motor delays (GMI scores < 70) and borderline impaired (GMI scores < 70 and 79) (Ulrich, 2019).

### Girls’ LOCS and BS motor trajectories across country comparisons

**Fig 2:**
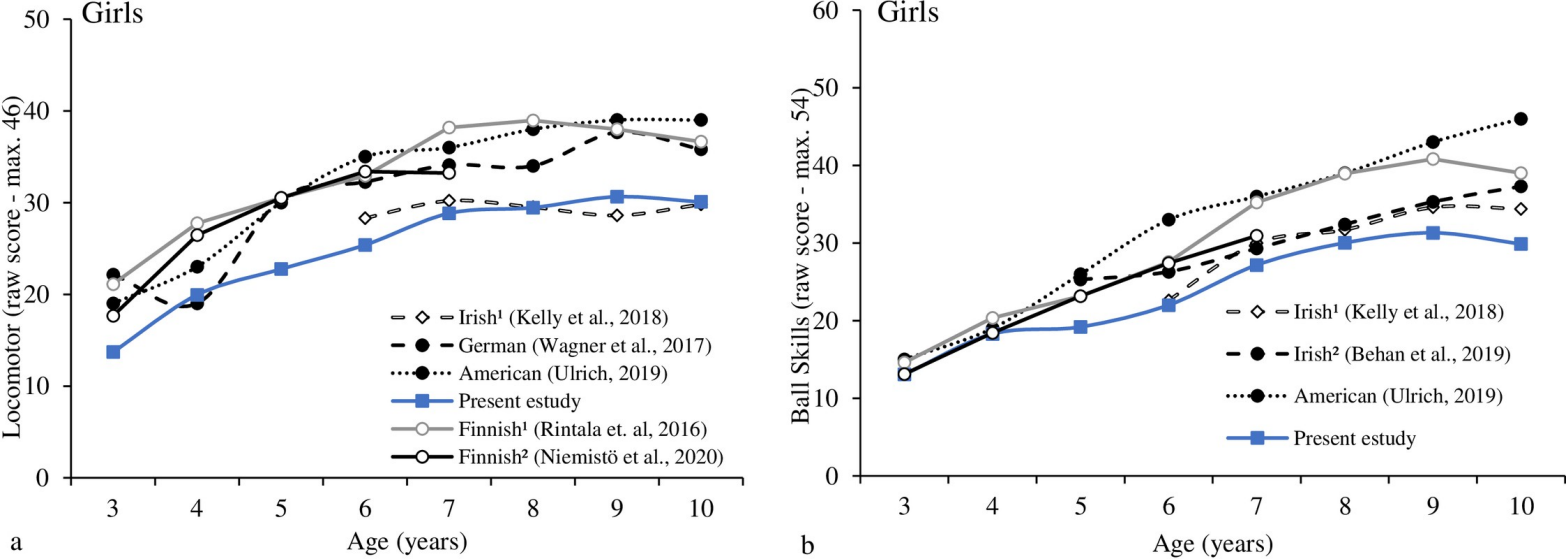
Motor trajectory for LOCS (2a) and BS (2b) for girls across countries.

Fig 2A shows LOCS scores for Brazilian, Irish, Finnish, German, and American girls. The results showed that Brazilian girls compared to Irish study 1 girls [3] had lower LOCS scores at 6-years-old (t = -2.6, p = .012) and higher at 9-years-old (t = 3.4, p = .001). Regarding the other countries, Brazilian girls showed significant lower LOCS scores in all age groups (t values between -12.8 and -11.2; p values between .002 and < .001), except at 4-years-old German girls (t = 1.11, p = .267).

Fig 2B shows BS scores for Brazilian, Irish study 1, Irish study 2, Finnish study 1, Finnish study 2, and American girls. The results showed lower BS for Brazilian girls than the Irish study 1 on 7-, 9-, and 10-years-old (t values between -3.57 and 2.57; p values between .001 and < .001). In addition, Brazilian girls showed lower BS than Irish study 2 in each age assessed (t values between -9.37 and -2.69; p values between < .001). Also, Brazilian girls showed lower BS than Finnish study 1 and American girls in each age assessed (t values between -16.45 and -2.22, p values between .032 and < .001). Exceptions were observed for Brazilian and Finnish girls (study 2) at 3- and 4-years-old and Brazilian and American girls at 4-years-old (t values between—.96 and .06, p values between .946 and .336). S1-S4 Tables in S1 File present all analysis results and are provided as supplementary material.

Table 2 provides the statistical results for the individual LOCS and BS comparisons across countries for girls. The results showed significantly higher scores for the Finnish girls than Brazilian girls for the run, hop, slide, jump, and two-hands strike from 3- to 10-years-old, with moderate to large effect size. Finnish girls also had higher scores on the gallop and one-hand strike, with small to large effect sizes, in different age groups. Brazilian girls showed higher scores than Finnish girls on catch from 3- to 5-years-old, with moderate to large effect sizes, and kick from 3- to 9-years-old, with moderate to large effect sizes. For skip, Brazilian girls at 3- and 6-years-old showed higher scores, whereas 4- and 7- to 10-years-old Finnish girls had higher scores than the other groups; small to moderate effect sizes were found. A similar trend was found for dribble, at 5-years-old Brazilian girls had higher scores, whereas from 6- to 10-years-old, Finnish girls had higher scores than Brazilians.

**Table 2:** Comparisons across WEIRD countries and Brazil: Statistical results (p and Cohen’s d) for the individual LOCS and BS for girls.

SKILLS	GIRLS: BR^1^ x FI^2^				GIRLS: BR^1^ x IRI^3^			
	Significant			Non-sig *p*>.050	Significant			Non-sig *p*>.050
	Age (years)	*P*	*d*	Age (years)	Age (years)	*p*	*d*	Age (years)
**Run**	3–10	< .001^FI^	.76 to 1.5	-	6–7 & 9–10	< .001^BR^	.66 to 1.5	8
**Gallop**	3 & 6–8	≤ .003^FI^	.36 to .61	4–5 & 9–10	9	< .001^BR^	.04	6–8 &10
**Hop**	3–10	< .001^FI^	.67 to 1.9	-	6	.003^IRI^	.37	7–10
**Skip**	3 & 6	≤ .023^BR^	.36 & .29	5	-	-	-	6–10
	4 & 7–10	≤ .046^FI^	.29 to .67					
**Slide**	3–10	< .001^FI^	.53 to .98	-	6 & 7	.001^IRI^	.52 & .25	8–10
**Jump**	3–10	< .046^FI^	.32 to 1.3	-	6–10	< .001^IRI^	.44 to 1.1	-
**Strike 2-hands**	3–10	< .001^FI^	.40 to 1.5	-	6–8	< .001^BR^	.47 to .72	9–10
**Strike 1-hand**	4–10	≤ .001^FI^	.47 to .89	3	6–7 & 9–10	≤ .003^IRI^	.48 to .51	8
**Dribble**	5	< .001^BR^	.41	3–4	6 & 9–10	< .001^IRI^	.54 to .75	7–8
	6–10	≤ .009^FI^	.34 to .70					
**Catch**	3–5	< .001^BR^	.41 to .81	6 & 8–10	6–10	≤ .002^BR^	.39 to 1.4	-
	7	< .001^FI^	.07					
**Kick**	3–9	≤ .009^BR^	.40 to .83	10	6–9	< .001^BR^	.79 to 1.0	10
**Underhand throw**	4–10	< .001^IRI^	.40 to 1.6	3	6–10	< .001^IRI^	.91 to 1.5	-
**Overhand throw**	3 & 5–10	< .001^FI^	.27 to 1.3	4	6–10	≤ .019^IRI^	.36 to .65	-

Note: ^1^ Present study;

^2^ Rintala et al. [44], 2016;

^3^ Kelly et al., 2018 [3];

Higher scores for: ^BR^ Brazilian girls;

^FI^ Finland girls;

^IRI^ Irish girls.

The analyses also showed higher scores for the Irish girls than Brazilian girls for jump and underhand throw and overhand throw from 6- to 10-years-old, with moderate to very large effect size. Irish girls also had higher scores on the hop, slide, one-hand strike, and dribble, with moderate effect sizes, in different age groups. Brazilian girls showed higher scores than Irish girls on the catch from 6- to 10-years-old and on the run, gallop, two-hands strike, and kick with large and very large effect sizes in some age groups.

**Fig 3:**
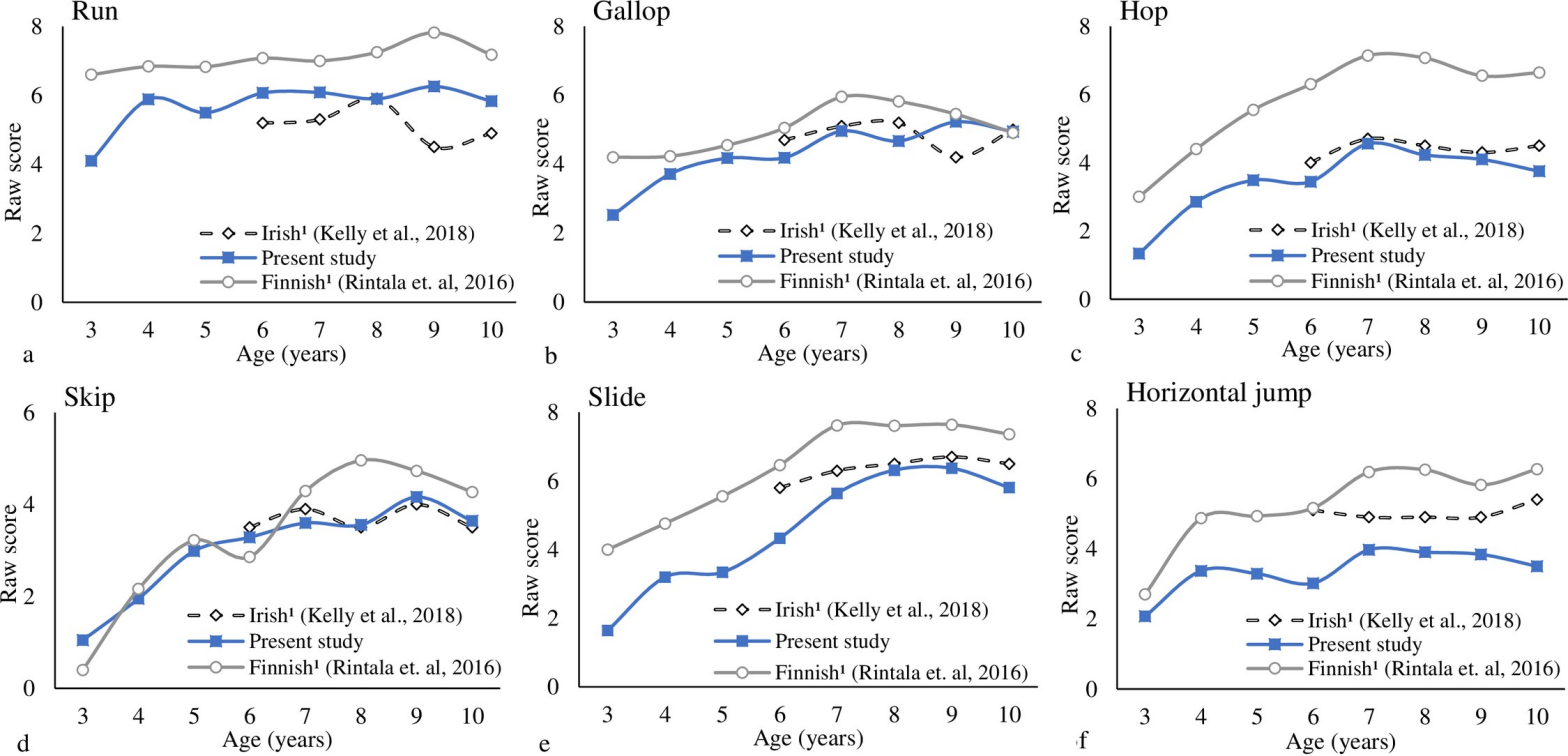
Individual locomotor skills trajectories for girls across countries (each skill maximum score is the reference).

**Fig 4:**
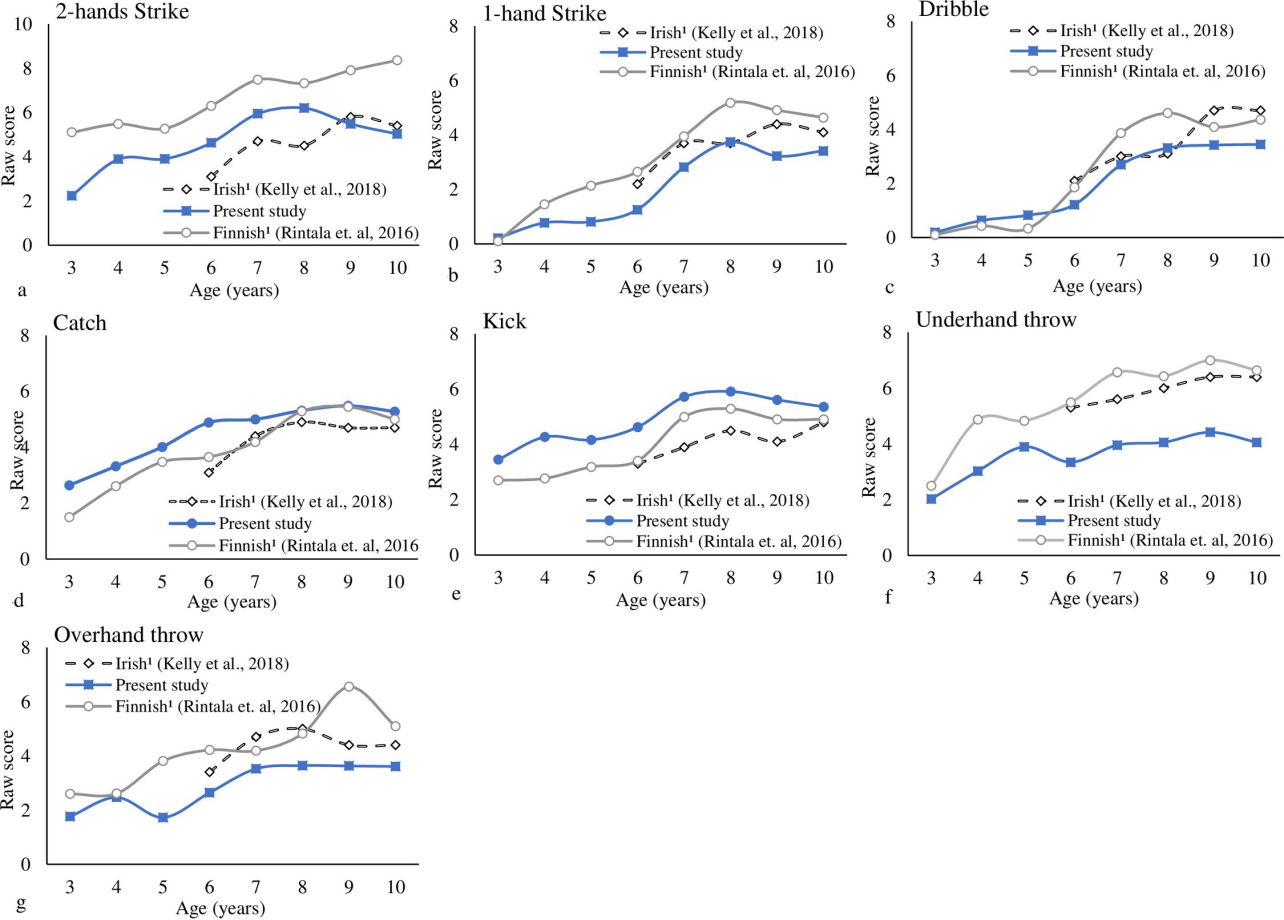
Individual ball skills trajectories for girls across countries (each skill maximum score is the reference).

Figs 3 and 4 provided the individual LOCS and BS trajectories for girls across countries. The development trajectory for the girls seems to achieve its peak around the age of 8- and 9-years-old for LOC and BS subtests, respectively. For all the samples, tiny increments were observed after that period, the only exception was the American girls on BS–increments were observed until 10-years-old, the ceiling effect was not observed in the samples. Regarding individual skills, no increments were observed after 8-years-old for any LOC skills; for the BS–two-hands strike with and overhand throw, some increments were observed from 8- to 10-years-old Finland girls.

### Boys LOCS and BS motor trajectories across country comparisons

**Fig 5:**
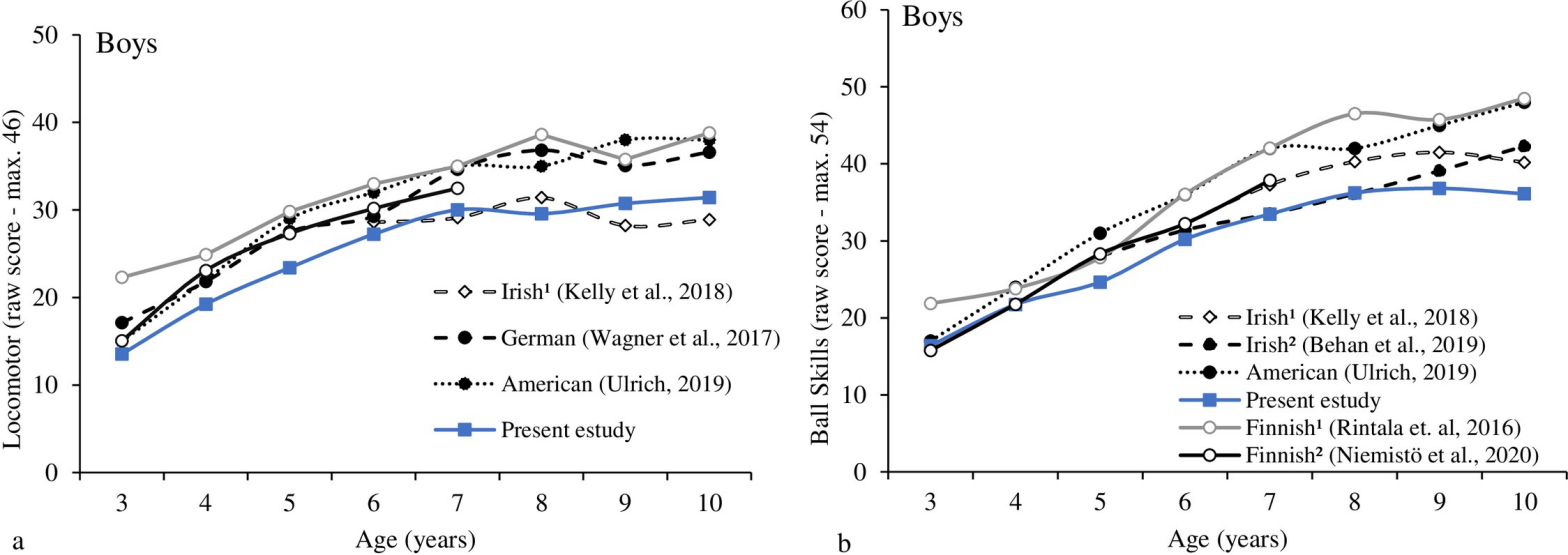
Motor trajectories for LOCS (5a) and BS (5b) for boys across countries.

Fig 5A shows the LOCS scores for Brazilian, Irish, Finnish (studies 1 and 2), German, and American boys. Compared to Irish, Brazilian boys showed lower LOCS scores at 8-years-old (t = -2.57, p = .012) and higher at 9 and 10-years-old (t values 3.41 and 3.67, p values = .001). Brazilian boys showed lower LOCS performance compared to German, Finnish (study 1), Finnish (study 2) and American (t values between -9.57 and -12.66, p values between .005 and < .001) in each age assessed; except at 3-years-old between Brazilian and American (t = -1.41, p = .167) and Brazilian and Finnish (study 2) (t = -1.46, p = .153). S1-S4 Tables in S1 File present all analysis results and are provided as supplementary material.

Fig 5B shows BS scores for Brazilian, Irish (studies 1 and 2), Finnish (studies 1 and 2), and American boys. Brazilian boys showed lower BS than Irish (study 1) in each age assessed (t values between -5.32 and -2.60; p values between .002 and < .001). Brazilian boys also showed lower BS than Irish (study 2) boys at 5, 9 and 10years-old (t values between -6.05 and -3.05; p values < .001). In addition, Brazilian boys also showed lower BS compared to Finnish (studies 1 and 2) and Americans in each assessed age (t values between -2.64 and -12.25; p values between .021 and < .001), except at 3- and 4-years-old between Brazilian and Finnish (study 2), and at 3-years-old, between Brazilian and Americans (t values between -.81 and -.04; p values between .964 and .442).

Table 3 provides the statistical test results for the individual LOCS and BS comparisons across countries for boys. In all age groups, the analyses showed significantly higher scores for the Finnish than Brazilian boys for the run, hop, jump, one-hand strike, two-hands strike, and the underhand throw, with moderate to very large effect size. Finnish boys also had higher gallop, slide, dribble, and overhand throw scores in different age groups, with small to large effect sizes. Brazilian boys showed a higher score than Finnish boys on the skip from 4- to 5-years and 10-years-old, dribble at the 4-years-old, catch from 3- to 5-years-old, and kick from 3- to 6-years old, with small to moderate effect sizes.

**Table 3:** Comparisons across WEIRD countries and Brazil: Statistical results (*p* and Cohen’s *d*) for the individual LOCS and BS for boys.

SKILLS	BOYS: BR^1^ x FI^2^				BOYS: BR^1^ x IRI^3^			
	Significant			Non-sig *p*>.050	Significant			Non-sig *p*>.050
	Age (years)	*P*	*d*	Age (years)	Age (years)	*p*	*d*	Age (years)
**Run**	3–10	≤ .023^FI^	.31 to 1.2	-	6–10	< .001^BR^	.54 to 1.0	-
**Gallop**	3 & 8	≤ .002^FI^	.51 & .52	4–7 & 9–10	8	< .001^IRI^	.48	6–7 & 10
					9	.011^BR^	.31	
**Hop**	3–10	< .001^FI^	.56 to 1.3	-	6, 8 & 10	≤ .023^IRI^	.18 to .53	7 & 9
**Skip**	4–5 & 10	≤ .043^BR^	.25 to 45	3, 6–7 & 9–10	9–10	≤ .038^BR^	.26 & .41	6–8
	8	.002^FI^	.38					
**Slide**	3 & 5–10	≤ .048^FI^	.25 to .80	4	6–9	≤ .002^IRI^	.42 & .75	10
**Jump**	3–10	< .001^FI^	.60 to 1.6	-	6–10	≤ .003^IRI^	.44 to .91	-
**Strike 2-hands**	3–10	< .001^FI^	.88 to 1.5	-	9–10	≤ .026^IRI^	.31 to .81	6–8
**Strike 1-hand**	3–10	≤ .003^FI^	.34 to 1.9	-	6–10	< .001^IRI^	.67 to .97	-
**Dribble**	6–10	≤ .007^FI^	.39 to 1.2	3 & 5	6–10	≤ .026^IRI^	.31 to .81	-
	4	.018^BR^	.02					
**Catch**	3–5	< .001^BR^	.38 to 1.4	6–10	6–10	≤ .046^BR^	.24 to 1.1	-
**Kick**	3–6	≤ .016^BR^	.59 to .94	7–8 & 10	6	.006^BR^	.40	7 & 9–10
	9	.022^FI^	.28		8	< .001^IRI^	.31	
**Underhand throw**	3–10	< .001^FI^	.82 to 1.9	-	6–10	< .001^IRI^	.75 to 1.4	-
**Overhand throw**	3–4 & 6–10	≤ .016^FI^	.38 to 1.2	5	6–10	< .001^IRI^	.69 to .95	-

Note: ^1^ Present study;

^2^ Rintala et al. [44], 2016;

^3^ Kelly et al., 2018 [3]; Higher scores for:

^BR^ Brazilian boys,

^FI^ Finland boys;

^IRI^ Irish boys.

The analyses also showed significantly higher scores for the Irish boys than Brazilian boys for jump, one-hand strike, dribble, and underhand throw and overhand throw from 6- to 10-years-old, with moderate to large effect size. Irish boys also had higher gallop, hop, slide, and two-hands strike scores in some age groups, with moderate to large effect sizes. Brazilian boys showed higher scores than Irish boys on the run and catch at most age groups, and on the skip in some age groups, with small to moderate effect sizes. Regarding the kick, Brazilian boys showed higher scores at 6-years-old whereas the Irish boys at 8-years-old.

**Fig 6:**
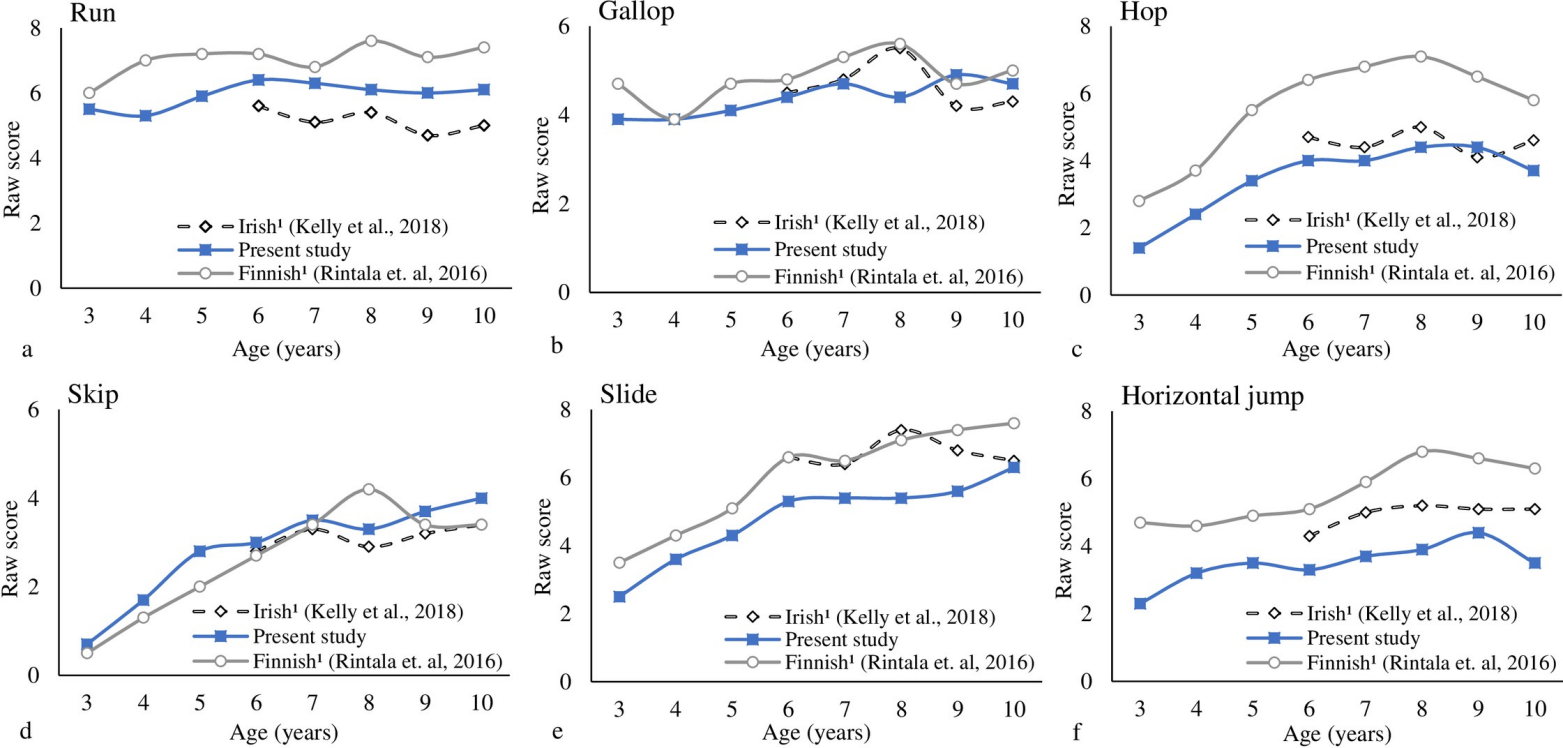
Individual locomotor skills trajectories for boys across countries (each skill maximum score is the reference).

**Fig 7:**
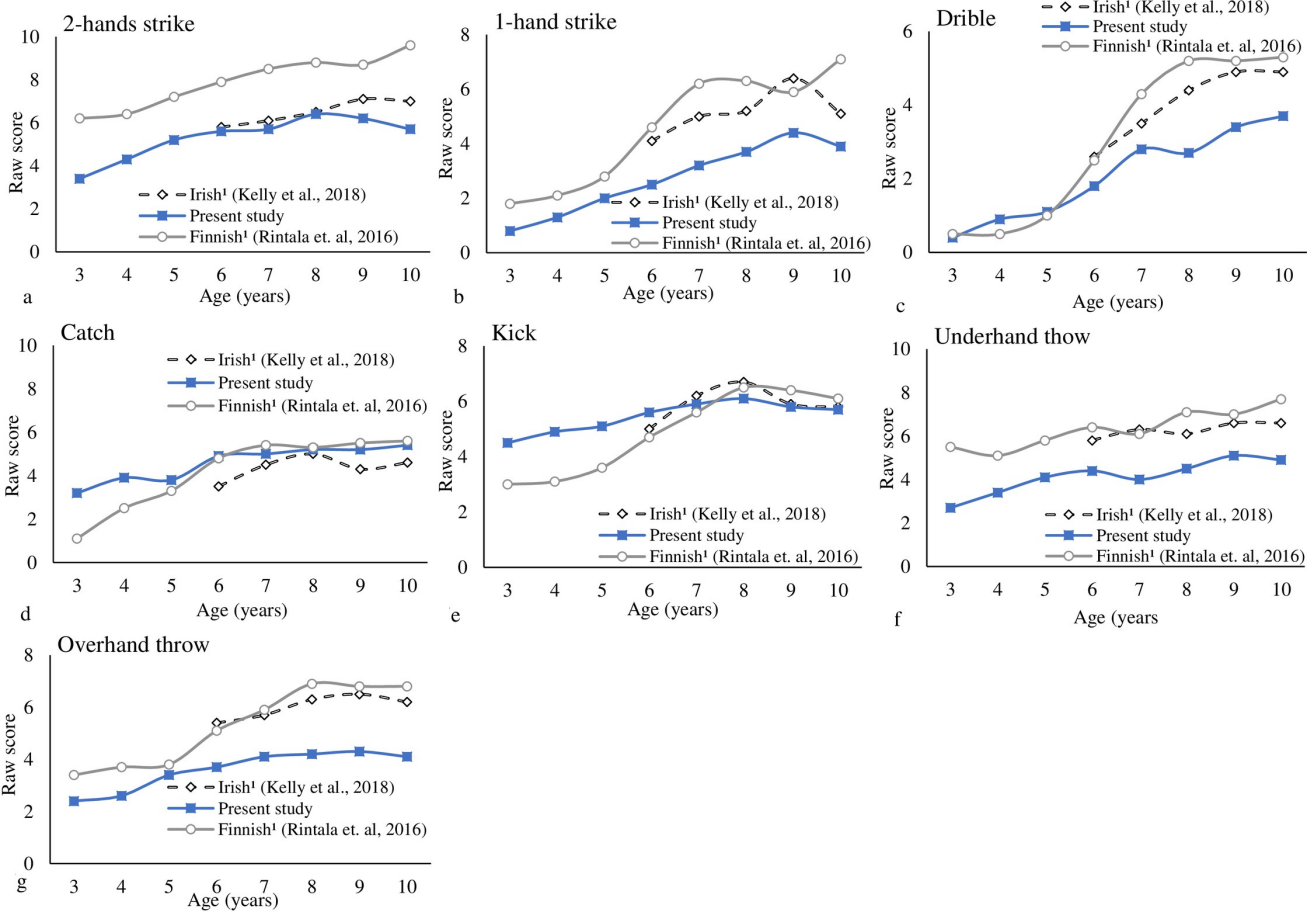
Individual ball skills for boys across countries (each skill maximum score is the reference).

Figs 6 and 7 provided the individual LOCS and BS trajectories for boys across countries. Overall, the development trajectory for the boys achieves its peak around 9- and 10-years-old for LOC and BS subtests, respectively. More variation was observed for LOCS; the samples did not show the ceiling effect. Some increments were observed for the skip from 8- to 10-years-old for Brazilian and Irish boys. For the one-hand strike and two-hands strike, and underhand throw, some increments were observed from 9- to 10-years-old for Finnish boys.

Please refer to S1-S4 Tables in S1 File for all statical results.

## Discussion

This study aimed to examine the prevalence of delays and borderline impaired performance for girls and boys in an LMIC country—Brazil, the variation in the motor trajectories (locomotor and ball skills) of girls and boys (3- to 10-years-old) across WEIRD countries, and Brazil, as well as the differences in the trajectories of specific skills for girls and boys across the countries. The evidence showed that Brazilian girls and boys had overall lower scores on the LOCS and BS subtest compared to Finnish, Irish, German, and American girls, as well as lower scores in most of the individual skills, compared to Finnish [44] and Irish [3] children, the only two samples that provided data for individual skills.

Overall, lower motor scores were observed for Brazilian girls and boys than the children in the WEIRD countries. Like previous Brazilian studies, the results indicated a higher prevalence of lower motor scores for Brazilian children across sex, age [5, 15, 19, 26], and socioeconomic status [31, 33, 37]. Children living in vulnerability had less opportunity to develop motor skills. It is essential to notice that SES is a crucial factor affecting children worldwide. Poverty has a striking influence on children due to the cumulative and prolonged exposure to risk factors, limiting the conditions for appropriate stimulation and access to opportunities that favor development outcomes at home and schools [33, 48–52]. Consequently, poverty underlays the other risk factors for adverse opportunities provided for children in Brazil and the consequent developmental outcomes [37, 53], and it may be the plausible explanation for the present result since 60% of children were from low-income families.

Regarding cross-cultural differences, the motor trajectories were distinct for the WEIRD countries compared to Brazil; overall, higher scores for LOCS and BS were observed for Americans, Finish, German, and Irish children than Brazilians. However, there were also exceptions; Brazilian children had a higher skip, catch, and kick scores than Finnish and Irish children. Compared to Finnish children, the advances for Brazilians were more prevalent up to 6-years-old, except for kick for girls–the higher scores found for Brazilian girls were from 3- to 9-years-old. Concerning Brazilian and Irish comparisons, the analysis was restricted to 6- to 10-years-old (the Irish study did not assess younger children), the Brazilian advances were observed from 6- to 10-years-old for most skills, the exception was the kick for boys that the advance for Brazilian’s boys was restricted to the age of 6-years-old.

Brazilian children’s experiences across childhood with dance, dodge games, and soccer practices at home and school may explain our results. Motor proficiency is supported by the exposure of the child to the family leisure activities at home [54], the home opportunities provided for the child [29, 55–57], the landscape in which the child play [55, 57, 58], the school curriculum [14, 59, 60], and the opportunities to engage in sports practices [14]. Our results support these contentions.

Among the after-school activities reported by the children in Brazil, soccer, and dance, respectively first and second, are the more frequent activities reported [14, 55, 57, 61]. The skip consists of a step and a hop, and its variants are part of several dance styles such as street, modern, ballet, and jazz. Kick is the focus of soccer, and soccer is the national sport in Brazil, played by children on every possible makeshift field [62], although it is still more prevalent among boys [14, 63]. Besides, dodge ball, known as "queimada" in Brazil, is a popular game and part of Brazil’s physical education curriculum [64]. The dodge game requires that the child catch a ball with different velocity, direction, and dislocation demands–features of proficient catching.

The means and variances for LOC and BS indicated a tendency, across all countries, for stabilization in scores at the age of 8- and 9-years-old for girls, whereas for boys at 9- and 10-years-old. No ceiling effects were found. The girls’ high means for LOCS were 7 points (American girls) to 16 points (Brazilian and Irish girls) below the maximum score in the test; and, for BS, 8 points (American girls) to 24 points (Brazilian girls). A similar trend was observed for boys; the boys’ high means for LOCS were 7 points (American and Finish boys) to 16 points (Irish boys) below the maximum score; and, for BS, 5 points (American and Finnish boys) to 18 points (Brazilian boys). Although motor performance differences prevailed among the investigated samples, moments of similar motor performances were across countries, more prevalent in the youngest ages (3- to 6-years-old). The low means and lack of celling effects lead us to inquire why children acquire several motor criteria for each skill but lack to acquire the more proficient ones. Further investigation is necessary to understand how children are being demanded to be more skilled within the context. In addition, if less instruction and feedback are provided to children after they acquire the essential motor criteria for a specific skill and become more independent to use the skill during games.

Another relevant aspect of this study was the observation of periods of stability in motor skill acquisitions, specifically after 8-years-old. This stability and instability in motor skill acquisition highlight the importance of monitoring the child’s motor trajectory to identify the actual motor changes and the periods of the crucial need for the teacher intervention to breach the proficiency barriers. However, the lack of individual skills means for several countries (US and Germany) limited, in part, the comparison of population samples and the discussion regarding the peculiarities of each skill. Besides, caution is necessary for interpreting these results since it is a cross-sectional design.

## Strengths and limitations

The strength of this study was to compare motor outcomes of girls and boys across diverse cultural contexts, WEIRD and LMIC countries, and provided evidence that children in LMIC(s) need further attention due to the lower scores in the motor trajectory across childhood. We also provided evidence that the cultural motor practices in Brazil (e.g., dance, soccer, dodge ball games) probably affect children’s performance since the only skills that Brazilian girls and boys showed consistently higher scores were skipped, kicking, and catching.

The results also highlight the need for further research to assess the exposure to different risk factors, biological (e.g., prematurity, exposure to drugs and heavy metals) and social (e.g., accesses to enrichment programs, leisure time, daily routine), or even different levels of risks between LMIC and WEIRD countries, and how it affects motor performances. Culture, family beliefs, curriculum, and opportunities to practice motor skills differ from LMIC and WEIRD countries; these factors also need further investigation—a limitation in the present study. Nevertheless, SES plays a relevant role in children’s opportunities to learn and acquire skills [36]. Although it was assessed in the Brazilian sample (60% of the children were from middle-low and poor), we have no information regarding families’ SES for the WEIRD countries’ studies, limiting our capability to analyze those differences further. Another limitation is the cross-sectional design; longitudinal data wild provided exciting insights into individual variations.

## Conclusion

In this cross-sectional study, the observed difference in gross motor trajectories for LOCS and BS of Brazilian children compared to WEIRD countries may have been determined by risk factors that increase vulnerability and predisposition to overall impairments from children from LMIC(s). Children from LMIC endure more health, nutrition, and social factors that prevent them from reaching their developmental potential; all aspects are emphasized by poverty. This exposure may have negative repercussions on the acquisition of motor skills.

This study is grounded in the understanding that countries’ differences or similarities in children’s performance and specific details about the actual differences across skills and age could lead to a collective effort to better understand motor trajectories across childhood in the light of each country’s singularities. A relevant part of the study was that engaging in critical inquiry about the Brazilian results compared to other countries could be a continuous learning process and possibly change the curriculum practices and improve children’s learning opportunities. Our results provided evidence that children from Brazil may need more extensive and cohesive support from schools and families and effective public policies to improve their motor competence. Here, we chose to point out the potential of a cross-cultural claim to challenge the status quo of children’s competence with international guidelines to develop motor skills across different cultures.

## Supporting information

S1 File Contains all the supporting tables. (DOCX)
