# Supplementary material for: Gross motor skills trajectory variation between WEIRD and LMIC countries: A cross-cultural study
Source: PLoS One. 2022 May 5;17(5):e0267665. doi: 10.1371/journal.pone.0267665 (PMC9070961; doi:10.1371/journal.pone.0267665)
Supplement: S1 File — (DOCX) [file pone.0267665.s001.docx]

Supplementary Tables

Table S1. Brazilian, US, Irish, German, and Finnish Boys results: One sample t test comparison for Locomotor skills

| **Locomotor** | **Boys** | | | | |
| --- | --- | --- | --- | --- | --- |
|  | BRA X US | BRA X IRI^1^ | BRA X GER | BRA X FIN^1^ | BRA X FIN^2^ |
| 3-years-old | t(34) = -1.41, p = 0.167 | - | t(34)= -3.46, p = 0.001 | t(34)= -8.48, p < 0.001 | t(34)= -1.46, p = 0.153 |
| 4-years-old | t(62) = -3.22, p = 0.002 | - | t(62)= -3.01, p = 0.004 | t(62)= -6.59, p < 0.001 | t(62)= -4.46, p < 0.001 |
| 5-years-old | t(74) = -7.25, p < 0.001 | - | t(74)= -5.36, p < 0.001 | t(74)= -8.39, p < 0.001 | t(74)= -5.08, p < 0.001 |
| 6-years-old | t(65) = -6.84, p < 0.001 | t(65)= -1.97, p = 0.053 | t(65)= -2.93, p = 0.005 | t(65)= -8.24, p < 0.001 | t(65)= -4.19, p < 0.001 |
| 7-years-old | t(78) = -6.10, p < 0.001 | t(78)= 1.11, p = 0.267 | t(78)= -5.67, p < 0.001 | t(78)= -6.10, p < 0.001 | t(78)= -3.00, p = 0.004 |
| 8-years-old | t(88) = -7.60, p < 0.001 | t(88)= -2.57, p = 0.012 | t(88)= -10.16, p < 0.001 | t(88)= -12.66, p < 0.001 | - |
| 9-years-old | t(74) = -9.78, p < 0.001 | t(74)= 3.41, p = 0.001 | t(74)= -5.82, p < 0.001 | t(74)= -6.75, p < 0.001 | - |
| 10-years-old | t(58) = -9.57, p < 0.001 | t(65)= -2.93, p = 0.005 | t(58)= -7.53, p < 0.001 | t(58)= -10.75, p < 0.001 | - |

Note: BRA: Brazilian children; US: American children; IRI^1^: Irish children (Kelly et. al., 2018); GER: German children; FIN^1^: Finnish children (Rintala et. al., 2016); FIN^2^: Finnish children (Niemisto et. al., 2020).

Table S2. Brazilian, US, Irish, German, and Finnish Boys results: One sample t test comparison for Ball skills

| **Ball skills** | **Boys** | | | | |
| --- | --- | --- | --- | --- | --- |
|  | BRA X US | BRA X IRI^1^ | BRA X IRI^2^ | BRA X FIN^1^ | BRA X FIN^2^ |
| 3-years-old | t(34)= -0.76 p = 0.451 | - | - | t(34)= -6.94 p < 0.001 | t(34)= 0.81 p = 0.422 |
| 4-years-old | t(62)= -2.58 p = 0.012 | - | - | t(62)= -2.36 p = 0.021 | t(62)= -0.45 p = 0.964 |
| 5-years-old | t(74)= -7.78 p < 0.001 | - | t(74)= -4.10 p < 0.001 | t(74)= -3.89 p < 0.001 | t(74)= -4.49 p < 0.001 |
| 6-years-old | t(65)= -7.53 p < 0.001 | t(65)= -2.60 p = 0.012 | t(65)= -1.56 p = 0.123 | t(65)= -7.62 p < 0.001 | t(65)= -2.64 p = 0.010 |
| 7-years-old | t(78)= -9.76 p < 0.001 | t(78)= -4.37 p < 0.001 | t(78)= -0.02 p = 0.983 | t(78)= -9.83 p < 0.001 | t(78)= -5.03 p < 0.001 |
| 8-years-old | t(88)= -6.70 p < 0.001 | t(88)= -4.73 p < 0.001 | t(88)= 0.14 p = 0.885 | t(88)= -11.95 p < 0.001 | - |
| 9-years-old | t(74)= -9.30 p < 0.001 | t(74)= -5.32 p < 0.001 | t(74)= -3.05 p = 0.003 | t(74)= -10.15 p < 0.001 | - |
| 10-years-old | t(58)= -11.75 p < 0.001 | t(58)= -4.03 p < 0.001 | t(58)= -6.11 p < 0.001 | t(58)= -12.25 p < 0.001 | - |

Note: BRA: Brazilian children; US: American children; IRI^1^: Irish children (Kelly et. al., 2018); IRI^2^: Irish children (Behan et. al., 2019); FIN^1^: Finnish children (Rintala et. al., 2016); FIN^2^: Finnish children (Niemisto et. al., 2020).

Table S3. Brazilian, US, Irish, German, and Finnish Girls results: One sample t test comparison of locomotor

| **Locomotor** | **Girls** | | | | |
| --- | --- | --- | --- | --- | --- |
|  | BRA X USA | BRA X IRI^1^ | BRA X GER | BRA X FIN^1^ | BRA X FIN^2^ |
| 3-years-old | t(39)= -4.38 p < 0.001 | - | t(39)= -6.97 p < 0.001 | t(39)= -6.12 p < 0.001 | t(39)= -3.26 p = 0.002 |
| 4-years-old | t(74)= -3.60 p = 0.001 | - | t(74)= -1.11 p = 0.267 | t(74)= -9.22 p < 0.001 | t(74)= -7.66 p < 0.001 |
| 5-years-old | t(72)= -10.56 p < 0.001 | - | t(72)= -11.20 p < 0.001 | t(72)= -11.30 p < 0.001 | t(72)= -11.33 p < 0.001 |
| 6-years-old | t(82)= -12.10 p < 0.001 | t(82)= -3.67 p < 0.001 | t(82)= -8.64 p < 0.001 | t(82)= -9.48 p < 0.001 | t(82)= -10.08 p < 0.001 |
| 7-years-old | t(80)= -9.80 p < 0.001 | t(80)= -1.86 p = 0.066 | t(80)= -7.18 p < 0.001 | t(80)= -12.80 p < 0.001 | t(80)= -6.02 p < 0.001 |
| 8-years-old | t(81)= -11.11 p < 0.001 | t(81)= 0.32 p = 0.745 | t(81)= -5.91 p < 0.001 | t(81)= -12.36 p < 0.001 | - |
| 9-years-old | t(81)= -12.13 p < 0.001 | t(81)= 2.97 p = 0.004 | t(81)= -10.20 p < 0.001 | t(81)= -10.68 p < 0.001 | - |
| 10-years-old | t(50)= -11.34 p < 0.001 | t(50)= 0.35 p = 0.725 | t(50)= -7.30 p < 0.001 | t(50)= -8.34 p < 0.001 | - |

Note: BRA: Brazilian children; US: American children; IRI^1^: Irish children (Kelly et. al., 2018); GER: German children; FIN^1^: Finnish children (Rintala et. al., 2016); FIN^2^: Finnish children (Niemisto et. al., 2020).

Table s4. Brazilian, USA, Irish, German, and Finnish Girls results: One sample t test comparison of Ball skills

| **Ball skills** | **Girls** | | | | |
| --- | --- | --- | --- | --- | --- |
|  | BRA X US | BRA X IRI^1^ | BRA X IRI^2^ | BRA X FIN^1^ | BRA X FIN^2^ |
| 3-years-old | t(39)= -2.80 p = 0.008 | - | - | t(39)= -2.22 p = 0.032 | t(39)= -0.06 p = 0.948 |
| 4-years-old | t(74)= -0.96 p = 0.336 | - | - | t(74)= -2.84 p = 0.006 | t(74)= -0.18 p = 0.852 |
| 5-years-old | t(72)= -10.44 p < 0.001 | - | t(72)= -9.37 p < 0.001 | t(72)= -6.10 p < 0.001 | t(72)= -6.05 p < 0.001 |
| 6-years-old | t(82)= -15.16 p < 0.001 | t(82)= -0.81 p = 0.420 | t(82)= -5.91 p < 0.001 | t(82)= -7.66 p < 0.001 | t(82)= -7.43 p < 0.001 |
| 7-years-old | t(80)= -11.16 p < 0.001 | t(80)= -3.57 p < 0.001 | t(80)= -2.69 p = 0.009 | t(80)= -10.20 p < 0.001 | t(80)= -4.76 p < 0.001 |
| 8-years-old | t(81)= -8.80 p < 0.001 | t(81)= -1.64 p = 0.104 | t(81)= -2.33 p = 0.022 | t(81)= -8.73 p < 0.001 | - |
| 9-years-old | t(81)= -12.22 p < 0.001 | t(81)= -3.42 p < 0.001 | t(81)= -4.16 p < 0.001 | t(81)= -9.94 p < 0.001 | - |
| 10-years-old | t(50)= -16.45 p < 0.001 | t(50)= -4.61 p < 0.001 | t(50)= -7.57 p < 0.001 | t(50)= -9.30 p < 0.001 | - |

Note: BRA: Brazilian children; US: American children; IRI^1^: Irish children (Kelly et. al., 2018); IRI^2^: Irish children (Behan et. al., 2019); FIN^1^: Finnish children (Rintala et. al., 2016); FIN^2^: Finnish children (Niemisto et. al., 2020).
